# Mechanomyography Signal Pattern Recognition of Knee and Ankle Movements Using Swarm Intelligence Algorithm-Based Feature Selection Methods

**DOI:** 10.3390/s23156939

**Published:** 2023-08-04

**Authors:** Yue Zhang, Maoxun Sun, Chunming Xia, Jie Zhou, Gangsheng Cao, Qing Wu

**Affiliations:** 1 School of Mechanical Engineering, Nantong University, Nantong 226019, China; yuezhang@ntu.edu.cn (Y.Z.);; 2 School of Mechanical Engineering, University of Shanghai for Science and Technology, Shanghai 200093, China; sunmaoxun@163.com; 3 School of Mechanical and Power Engineering, East China University of Science and Technology, Shanghai 200237, China; cmxia@ecust.edu.cn (C.X.);

**Keywords:** feature selection, chameleon swarm algorithm, grasshopper optimization algorithm, mechanomyography, pattern recognition

## Abstract

Pattern recognition of lower-limb movements based on mechanomyography (MMG) signals has a certain application value in the study of wearable rehabilitation-training devices. In this paper, MMG feature selection methods based on a chameleon swarm algorithm (CSA) and a grasshopper optimization algorithm (GOA) are proposed for the pattern recognition of knee and ankle movements in the sitting and standing positions. Wireless multichannel MMG acquisition systems were designed and used to collect MMG movements from four sites on the subjects thighs. The relationship between the threshold values and classification accuracy was analyzed, and comparatively high recognition rates were obtained after redundant information was eliminated. When the threshold value rose, the recognition rates from the CSA fluctuated within a small range: up to 88.17% (sitting position) and 90.07% (standing position). However, the recognition rates from the GOA drop dramatically when increasing the threshold value. The comparison results demonstrated that using a GOA consumes less time and selects fewer features, while a CSA gives higher recognition rates of knee and ankle movements.

## 1. Introduction

For diseases such as stroke, if patients do not realize the importance of rehabilitation training or cannot persist in it because of insufficient enthusiasm, their body function will deteriorate in the long term, negatively affecting their quality of life. Reasonable rehabilitation, especially active training with another person can have a positive effect on the recovery of the neuromuscular system and prevent palindromia or sequela. Thus it is necessary to study accurate and real time recognition method of human limb movements for rehabilitation training with human machine interaction.

Currently, biomedical signals, especially surface electromyography (sEMG), have been widely used in the pattern recognition of lower limb movements, and great progresses has been made in fields such as exoskeleton rehabilitation training, human machine interaction (HMI) systems and prosthetics [1]. Ryu et al. [2] proposed a method to extracted top and slope (TAS) features of sEMG to detect human lower movements. This method can reflect the time characteristic of sEMG and improve average detective accuracy. Yao et al. [3] extracted time-domain and frequency features of an 8-channel sEMG and adopted a support vector machine (SVM), extreme learning machine (ELM) and deep neural network (DNN) to achieve the recognition of 6-class gait movements. Shi et al. [4] used principal component analysis (PCA) to reduce the dimensionality after extracting features based on wavelet transform and then proposed a novel method to achieve the recognition of 5-class lower limb movements. He et al. [5] proposed a sparrow search algorithm-optimized deep belief network (SSA-DBN) classification method of gait movements after extracting wavelet features. The results demonstrated that the SSA effectively improved the performance of the DBN. Qin and Shi [6] extracted 11 features of three-channel sEMG and adopted a multikernel-relevant vector machine to achieve the recognition of 5-class upper-limb movements with a recognition rate up to 96%. In another study on the pattern recognition of upper limb movements, Wei et al. [7] proposed a method based on tunable Q-factor wavelet transform (TQWT) and Kraskov entropy, and achieved a high recognition rate of 4-class lower limb movements.

In contrast to sEMG, mechanomyography (MMG) is a low-frequency mechanical signal generated from muscle’s lateral oscillation and has application value in human machine interaction technology. Compared with sEMG, MMG is more convenient for collecting since it is unaffected by sweating and can be collected through specific cloth material [8,9]. Yu et al. [10] collected 4-channel MMG from the thigh and adopted a hidden Markov model to achieve recognition of gait movements. In another study, a Salp Swarm Algorithm (SSA) was used for time-domain feature extraction, and obtained better recognition results compared with some traditional feature-selection algorithms [11]. Nowadays, deep learning has been made great progress in image and natural-language processing and has been applied in the classification of biomedical signals with good performance [12,13,14], especially for big data. Since deep learning needs hardware with a higher configuration and takes a much longer time to train models, machine learning still has a certain application value if a simple model can perform the recognition task. Therefore machine learning is still worth investigating in pattern recognition of limb movements based on MMG.

When studying machine learning for biomedical signals, feature extraction and selection are two factors that directly affect classification accuracy. So far, time-domain [15,16], frequency-domain [17,18], and time-frequency-domain features [19,20] have been increasingly applied in the machine learning of sEMG, and nonlinear dynamic analysis features [7] have been used in the pattern recognition of human movements. If the dimension of the feature set is too high, the structure of the trained model will be overly complicated. Thus, an effective method for selecting features should be found before the features are input into the classifiers. Currently, some filter-feature selection methods, such as ReliefF and Laplacian, have been adopted to investigate the pattern recognition of human movement based on sEMG [21]. These methods have high efficiency, but they are not classification algorithms, and this may lead to a comparatively lower quality of selected features for a specific classification algorithm. Unlike the filter-feature selection method, a heuristic searching algorithm can adopt swarm intelligence, which simulates the model to use local information to generate unpredictable swarm behavior. The advantage is that the solution of a whole problem does not depend on just one part, giving the model stronger robustness and globally optimizing the problem in many situations. On the one hand, some swarm intelligence algorithms (SIAs) have so far been used to explore the pattern recognition of human movement based on biomedical signals by optimizing some classification algorithms [22,23]. On the other hand, an SIA can be used as the optimization algorithm of feature selection, i.e., taking the results of feature selection as the design variables as has been achieved in some fields [24,25]. Thus, feature-selection methods based on SIAs are worth being studied for biomedical signals. The main contribution of this paper is that MMG feature selection methods based on a chameleon swarm algorithm (CSA) and grasshopper optimization algorithm (GOA) are proposed for investigating the pattern recognition of knee and ankle movements, analyzing the relationship between threshold values and classification accuracy, and obtaining comparatively high recognition rates provided that redundant information is eliminated.

## 2. Materials and Methods

### 2.1. Experiment Protocol and Data Preprocessing

**Figure 1:**
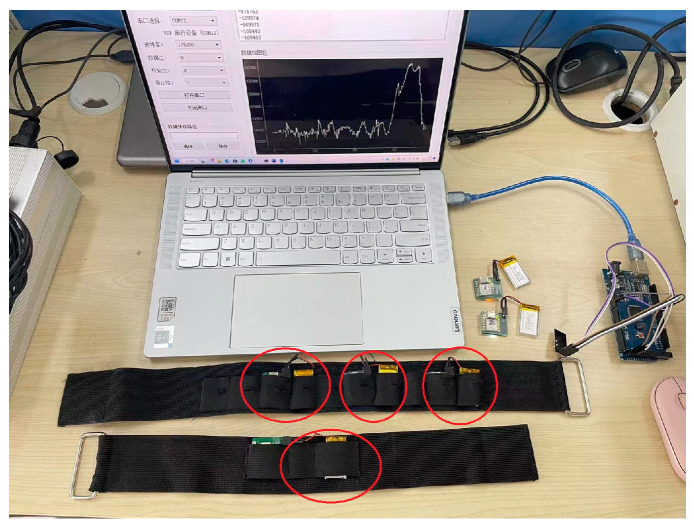
Acquisition system with 4 channels.

**Figure 2:**
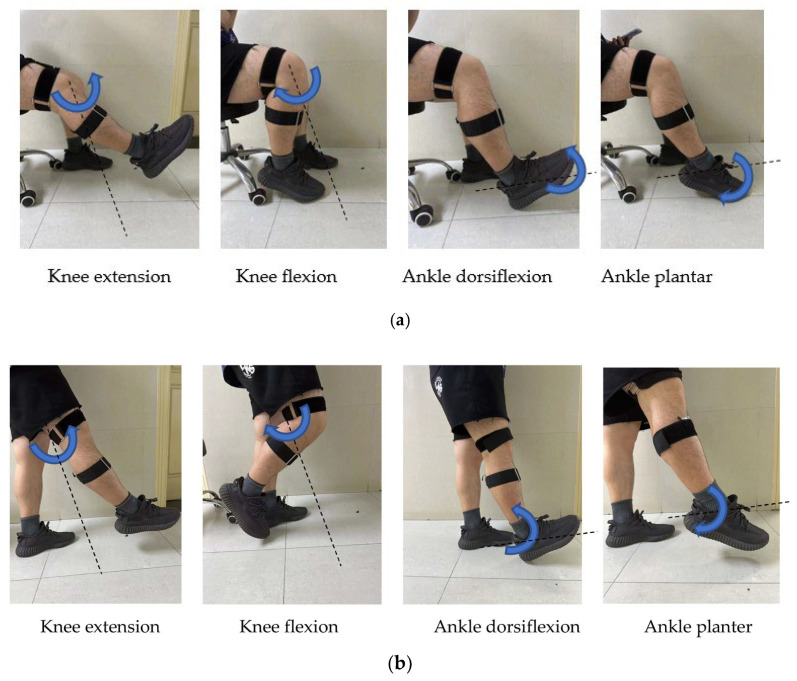
(**a**). Four classes of knee and ankle movement in the sitting position. (**b**)**.** Four classes of knee and ankle movement in the standing position.

This experiment was approved by the Ethics Committee of East China University of Science and Technology (Number 001/2019). In the experiment, seven healthy subjects (students from East China University of Science and Technology aged 23 to 26) who had no history of lower-limb muscle disorder and who had not done strenuous exercise within 48 h before the experiment were recruited. They agreed with the content of experiments and gave informed consent. An acquisition system each with a module integrated by MMG sensors (ADXL355, Analog Device, Inc., Norwood, MA, USA)—Seeeduino Xiao microprocessor (Seeed Technology, Co., Ltd, Shenzhen, China), power-supply with 3.7 V and 400 mAh (PULAN Technology, Co., Ltd., Hong Kong, China) and wireless-transmission units nRF24L01 (Nordic Semiconductor, Co., Ltd, Stockholm, Sweden)—was designed to collect MMG, shown as Figure 1. Since the main frequency band of MMG is lower than 100 Hz, the sampling rate was set at 250 Hz. Four sites on the thigh, i.e., vastus lateralis, vastus medialis, gastrocnemius lateralis and gastrocnemius medialis were selected for MMG collection, recorded as Channel 1 to 4. The experiment was divided into two cases, as shown in Figure 2. In Case 1, the subjects performed four class movements: knee extension (KE1), knee flexion (KF1), ankle dorsiflexion (AD1) and ankle plantar (AP1) in a sitting position, as shown in Figure 2a. In Case 2 the subjects performed four class movements: knee extension (KE2), knee flexion (KF2), ankle dorsiflexion (AD2) and ankle plantar (AP2) in a standing position, as shown in Figure 2b. They performed the knee and ankle movements without muscular discomfort. They performed the movements in time to a metronome in a quiet environment. To avoid muscle fatigue and hampering accurate performance, the movements of each class were repeated 100 times with plenty of rest between movements of different classes. Based on above the conditions, MMG was obtained from the standard movements.

**Figure 3:**
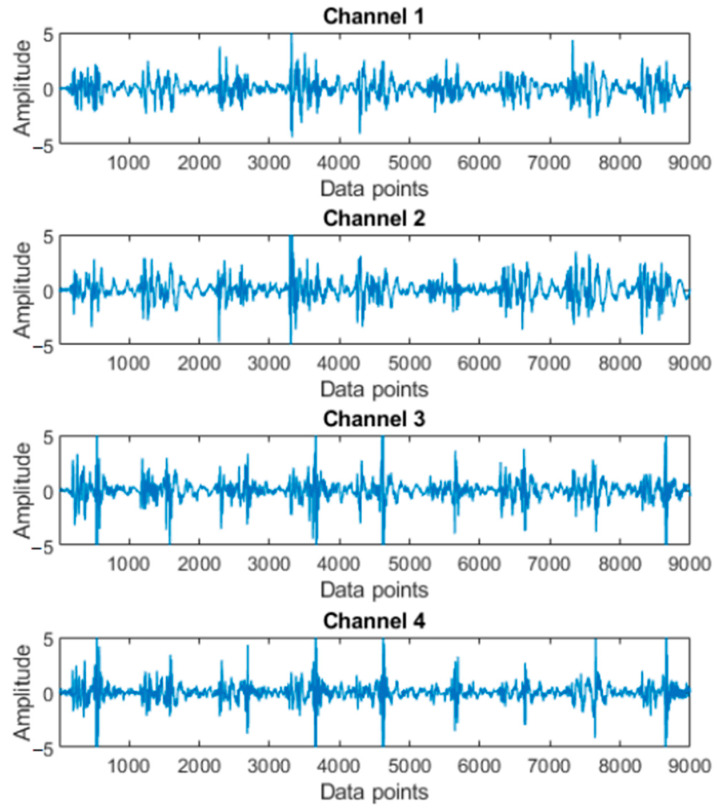
MMG signals with filtering and normalization.

**Figure 4:**
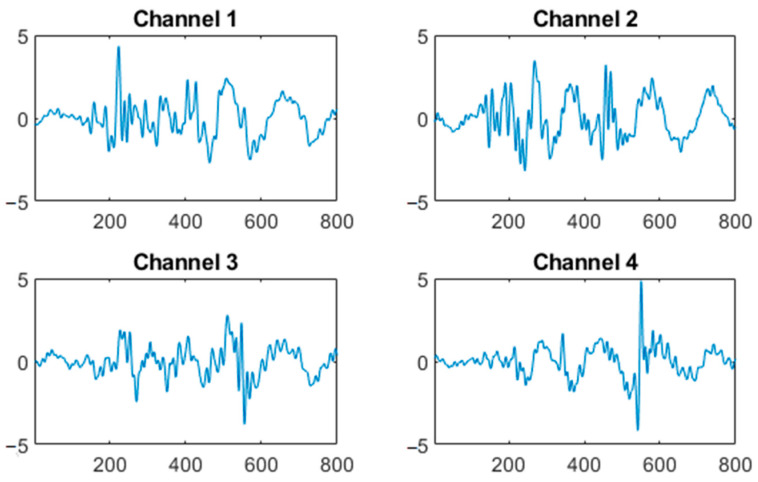
Four-channel MMG of one ankle dorsiflexion motion.

According the characteristics of MMG, a 5–100 Hz band-pass digital filter was used to process the signals, retaining useful information and removing the motion artifact [26]. The processed MMG signals are shown as Figure 3. Then the signals were segmented by the short-time energy method according each movement. In particular, the signals were divided into data blocks by a moving window. The average energy was calculated from the data blocks of all the channels, and then the start and end situations were found according to a threshold. Finally, the signal segments were obtained [27]. The MMG signals of a movement are shown in Figure 4.

### 2.2. Feature Extraction

#### 2.2.1. Time Domain Features

Since the signals can be regarded as a function of time, time-domain (TD) features can be calculated directly from a one-dimensional signal. Ten time-domain features [28]—root mean square (RMS), variance (VAR), zero crossing (ZC), wavelet length (WL), slope sign change (SSC), log detector (LOG), mean absolute value (MAV), v-order, simple square integral (SSI) and average amplitude change (AAC)—were extracted from all signals.

#### 2.2.2. Frequency Domain Features

Frequency domain (FD) features are indexes that analyze signals in the frequency domain. It can be calculated based on the power spectrum density (PSD), which is the power function of the frequency component in the unit bandwidth power. Two regularly used features—mean power frequency (MPF) and median frequency (MF) [29]—were adopted in this paper.

#### 2.2.3. Wavelet Packet Node Energy

Time-frequency analysis uses the joint distribution function of time and frequency to represent a signal, and wavelet analysis is a common method in biomedical signal processing [24]. After MMG was decomposed by a wavelet packet, a set of wavelet coefficients was obtained:

(1) $$S(t)=\sum_{j=0}^{2^{i}-1}f_{i,j}(t_{j})=f_{i,0}(t_{0})+f_{i,1}(t_{1})+\cdots f_{i,j}(t_{j})$$

where $f_{i,j}(t_{j})$ is the reconstructed signal of MMG node (i,j) decomposed by a wavelet packet. After calculating the node energy according to Parseval’s theorem, the energy of the ith level signal was defined as

(2) $$E_{i,j}(t_{j})=\sum_{k=1}^{m}{\left|x_{j,k}\right|}^{2}$$

where $E_{i,j}(t_{j})$ is the energy of frequency band in the jth node of the ith level signal; m is the number of the sampling data; and $x_{j,k}$ is the amplitude of the discrete point of the reconstructed signal. A four-level wavelet packet decomposition was adopted, and the energy features of 11 nodes corresponding to 7.8–93.8 Hz were extracted.

#### 2.2.4. Nonlinear Dynamic Analysis Feature

A human biomedical signal has obvious nonlinear characteristics; thus, some features based on a nonlinear dynamic (NLD) analysis—approximate entropy (AEn), sample entropy (SEn) and fuzzy entropy (FEn)—were used in the study of sEMG [30]. However, the computational requirements of NLD features are much more demanding, so they were adopted comparatively less in HMI systems based on sEMG. Lempel Ziv complexity (LZC) was used to represent the time series by symbol, and it increased with the disorder degree of signals. In this paper, AEn, SEn, FEn and LZC were extracted from each signal segment.

### 2.3. Swarm Intelligence Algorithms

#### 2.3.1. Chameleon Swarm Algorithm

A chameleon swarm algorithm (CSA) [31] is a kind of novel meta-heuristic optimization algorithm based on chameleon predation. It solves optimization problems mainly according to updated positions from the following three stages: searching for the prey, moving the eyes to find the prey; and catching the prey.

Step 1: Initialize the population size N, the position $X_{i}$, and the maximum iteration times $T_{\max}$. The initial positions of the chameleons can be described as

(3) $$X_{ij}=Ub+r\times (Ub-Lb)$$

where $x_{ij}$ is the position of the jth dimension of the ith chameleon; Ub and Lb are the upper bound and lower bound of the searching space; r is a random number between (0, 1).

Step 2: During the predation stage, the chameleon swarm searches for prey by updating their positions. The updated positions can be described as

(4) $$\left\{\begin{array}{l} X_{ij}^{t+1}=X_{ij}^{t}+p_{1}r_{2}(P_{ij}^{t}-G_{j})+p_{2}r_{1}(G_{j}-x_{ij}^{t}),r\geq Pp\\ X_{ij}^{t+1}=X_{ij}^{t}+\mu (r_{3}(Ub-Lb)+Lb)\mathrm{sgn}(rand-0.5),r<Pp \end{array}\right.$$

where $X_{ij}^{t}$ is the iteration position of the jth dimension of the ith chameleon; $p_{1}$ and $p_{2}$ are the control parameters of developing ability; $r_{1}$ and $r_{2}$ are the random numbers between (0, 1); $P_{ij}^{t}$ is the global optimization position of the jth dimension of the ith chameleon; Pp is the perceptual parameter; μ is the control parameter of searching ability, $\mu =e^{{\left(-\alpha t/T\right)}^{3}}$—α is the sensitivity coefficient; T is the maximum iteration number and t is the current iteration number.

Step 3: The chameleon finds the prey by moving its eyes and updating its own position according to the location of the prey. The updated position can be described as

(5) $$X_{i}^{t+1}=m\times (X_{i}^{t}-{\bar{X}}_{i}^{t})+{\bar{X}}_{i}^{t}$$

where $X_{i}^{t}$ is the iteration position of the ith chameleon; ${\bar{X}}_{i}^{t}$ is the central position of the ith chameleon; and m is the rotation matrix.

Step 4: When the prey is near, the chameleon will use its tongue to catch the prey. The velocity of movement can be described as

(6) $$\left\{\begin{array}{l} v_{i,j}^{t+1}=\omega v_{i,j}^{t}+c_{1}r_{1}(G_{j}-X_{ij}^{t})+cr(P_{ij}^{t}-X_{ij}^{t})\\ \omega ={\left(1-t/T\right)}^{p\sqrt{t/T}} \end{array}\right.$$

where $v_{ij}^{t+1}$ is the velocity of the jth dimension of the ith chameleon; $c_{1}$ and $c_{2}$ are two constant numbers; $r_{1}$ and $r_{2}$ are random numbers between (0, 1); and ω is the linearly decreasing inertial weight.

The updated position can be described as

(7) $$X_{ij}^{t+1}=X_{ij}^{t}+[{\left(v_{ij}^{t}\right)}^{2}-{\left(v_{ij}^{t-1}\right)}^{2}]/2a$$

where a is the acceleration and $a=2590\times (1-e^{-lgt})$.

#### 2.3.2. Grasshopper Optimization Algorithm

Grasshopper optimization algorithm (GOA) [32] is one other kind of novel meta-heuristic optimization algorithm, which is based on foraging of grasshopper.

Step 1: Initialize the population size N, the position $X_{i}$, the maximum iteration times $T_{\max}$, the range $c_{\max}$ and $c_{\min}$. The position of the ith individual is

(8) $$X_{i}=S_{i}+G_{i}+A_{i}$$

where $S_{i}$ is the effect on the ith individual from the population; $G_{i}$ is the effect from gravity; and $A_{i}$ is the effect from wind.

To solve the optimization problems, $G_{i}$ and $A_{i}$ were often replaced with the position of targeted food $T_{d}$, thus

(9) $$X_{i}=S_{i}+T_{d}$$

(10) $$S_{i}=\sum_{j=1,j\neq i}^{N}s(\left|x_{j}-x_{i}\right|)\frac{x_{j}-x_{i}}{\left|x_{j}-x_{i}\right|}$$

where s(r) is the interaction force and it is defined as

(11) $$s(r)=fe^{-\frac{r}{l}}-e^{-r}$$

where the individuals attract each other when s>0, and exclude each other when s<0. Here f is the attractive factor, and l is the attractive scale.

Step 2: Calculate the fitness function value of each grasshopper.

Step 3: Update the control parameter c of the searching space, which can be described as

(12) $$c=c_{\max}-t\frac{c_{\max}-c_{\min}}{T_{\max}}$$

where t is the current iteration time, and $T_{\max}$ is the maximum iteration time.

Step 4: The updated position of grasshopper can be described as

(13) $$X_{i}^{d}=c\sum_{j=1,j\neq i}^{N}c\frac{Ub-Lb}{2}s(\left|x_{j}-x_{i}\right|)\frac{x_{j}-x_{i}}{\left|x_{j}-x_{i}\right|}+T_{d}$$

where Ub and Lb are the upper and lower bounds of the searching space, and $T_{d}$ is the best solution of the swarms in the space.

### 2.4. Classification Algorithm

As a kind of algorithm with good performance in high dimension and nonlinear classification problems, a support vector machine (SVM) was used to build the classifier. If a set with two classes of data $\left\{x_{i},y_{i}\right\}$ (i=1,2,⋯,N, $y_{i}\in \{-1,+1\}$, $x_{i}\in R^{d}$) were given, the slack variable $\xi_{i}\geq 0$ and penalty parameter C>0 were introduced, and then the optimal hyperspace was

(14) $$\max\sum_{i=1}^{m}\alpha_{i}-\frac{1}{2}\sum_{i=1}^{m}\alpha_{i}\alpha_{j}y_{i}y_{j}(x_{i},x_{j}),\;s.t.\;0\leq \alpha_{i}\leq C,\;\sum_{i=1}^{m}\alpha_{i}y_{i}=0,\;i=1,\cdots ,m$$

where $\alpha_{i}$ is the Lagrange factor.

The kernel function was introduced to increase the dimension, thereby obtaining the optimal hyperspace as $f(x)=sign[\sum_{i=1}^{N}\alpha_{i}y_{i}K(x_{i}\cdot x)+b]$. Some kernel functions such as the radial basic function (RBF) had the characteristic of a simple structure and high generalization performance. The formula of RBF is

(15) $$K(x,x’)=\exp(-\frac{{\left\|x-x’\right\|}^{2}}{\sigma^{2}})$$

In some studies about the classification of sEMG, using a SVM with RBF can achieve better results than using K nearest neighbor (KNN), linear discriminant analysis (LDA), random forest (RF) or some other classification algorithms [33,34].

### 2.5. Feature Selection

**Figure 5:**
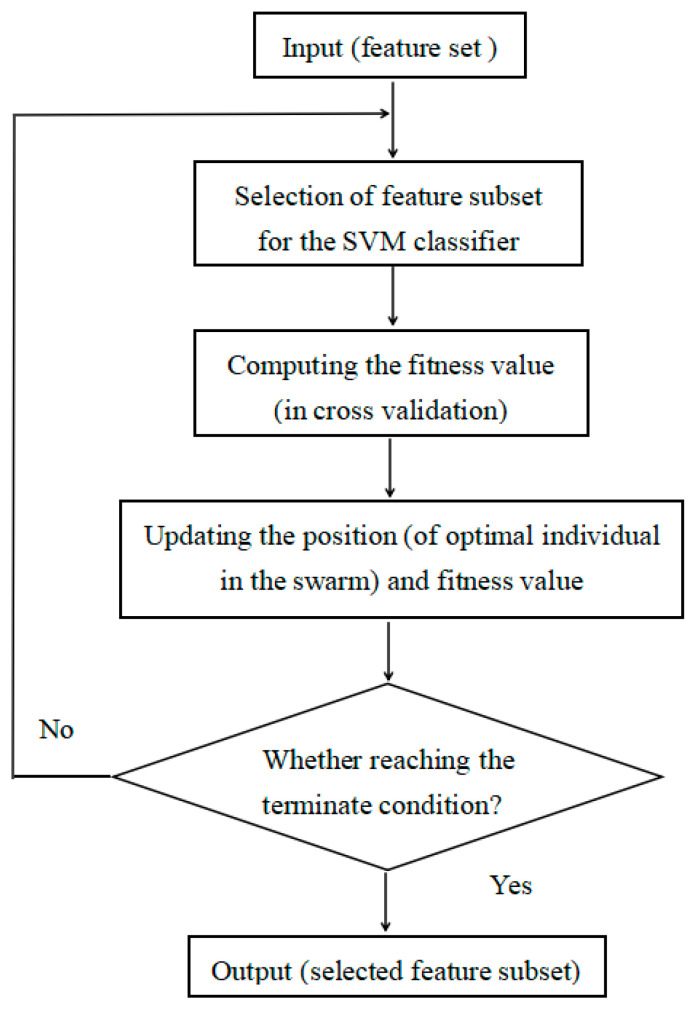
Flow chart of feature selection based on swarm intelligence algorithms.

Feature selection can be used to obtain important and representative features from the set, reducing the dimension of feature set. For the optimization problem, classification accuracy was taken as the target function f(X) to evaluate the effect of feature selection. In this paper, CSA and GOA were used as two searching algorithms to derive an optimal feature subset. Each individual represented a solution, and its position information $X_{i}$ was taken as the design variable for the optimal problem. The value of $X_{i}$ was changed by a transfer function and compared with a threshold T to decide if this feature were retained. The flow chart of feature selection based on SIAs is shown in Figure 5.

Several functions can be adopted as the transfer function as shown as Table 1. In this paper, S1 was adopted. In the process of iteration, the position of an individual was updated according to the fitness function. When the classification accuracy was higher and the number of selected features was lower, the result of the feature selection was meaningful. The fitness function was defined as

(16) Fitness=α⋅acc+β(1−R/N), β=1−α

where acc is the classification accuracy; R and N are the numbers of selected features and all features, respectively; and α is 0.99.

The updated positions were changed by the transfer function, and then the values were compared with the threshold T. If the values were larger than T, the corresponding features were selected; otherwise, the corresponding features were not selected. The classification results of the models were obtained based on intra-subject since it was impossible to avoid the differences between subjects. A 10-fold cross validation was applied to evaluate the performance of the classifier and the effect of feature selection. The final results are shown as Equations (2)–(17) and Equations (2)–(18). The samples of every subject were divided into 10 folds, and 1 fold from each was retained as a testing set; the other 9 were used for training a model.

(17) $$mean\_acc=\frac{1}{K}\sum_{i=1}^{K}acc(i)$$

(18) $$mean\_R=\frac{1}{K}\sum_{i=1}^{K}R(i)$$

## 3. Results

### 3.1. Classification Results from Different Feature Set

**Figure 6:**
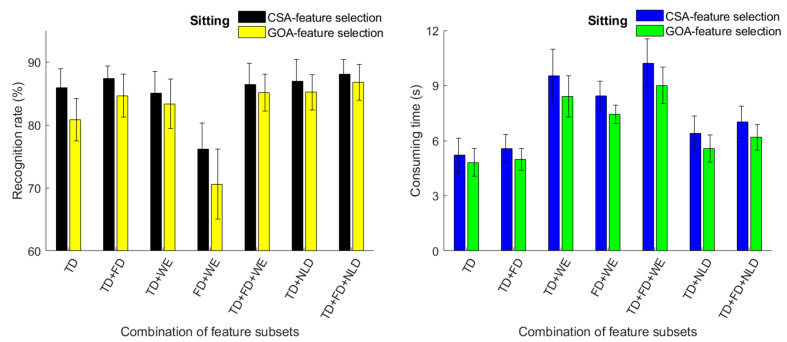
Classification accuracy (%) of four-class movements and time consumption (s) of feature selection (sitting situation).

**Figure 7:**
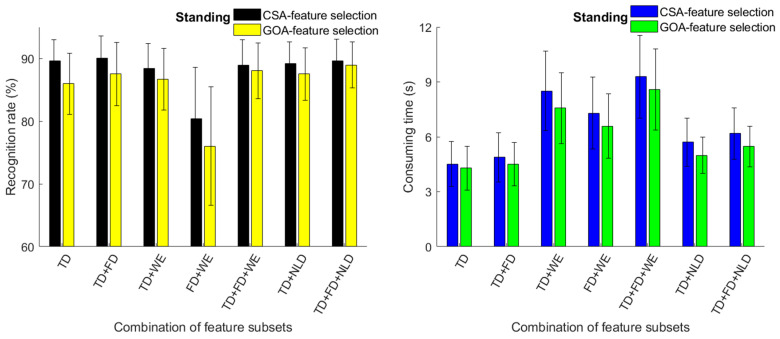
Classification accuracy (%) of four-class movements and time consumption (s) of feature selection (standing situation).

To evaluate the effect on movement classification from different feature sets, 7 feature set were constructed from TD, FD, WE and NLD features. For two cases (sitting and standing), the classification accuracy and time consumption are shown in Figure 6 and Figure 7. Among all the feature sets, using TD + FD + NLD obtained better results. For the CSA, the classification accuracy was 88.43% for movement in a sitting position and 89.73% for movements in a standing position; there was no significant difference (*p* > 0.05). For the GOA, the classification accuracy was 86.87% for sitting and 89.01% for standing; there was also no difference significant difference (*p* > 0.05). In the situation with a specific T, using the CSA to select features achieved a higher classification accuracy than using the GOA. TD features played an important role in high classification accuracy since using FD + WE (without TD) merely obtained an accuracy of 70.59% (sitting) and 76.02% (standing), much lower than using other feature sets (sitting: vs. TD, *p* < 0.01; vs. TD + FD, *p* < 0.01; vs. TD + WE, *p* < 0.01; vs. TD + FD + WE, *p* < 0.01, vs. TD + NLD, *p* < 0.01; vs. TD + FD + NLD, *p* < 0.01.) (standing: vs. TD, *p* < 0.05; vs. TD + FD, *p* < 0.05; vs. TD + WE, *p* < 0.05; vs. TD + FD + WE, *p* < 0.05; vs. TD + NLD, *p* < 0.05; vs. TD + FD + NLD, *p* < 0.01.). Concerning average time consumption, using a TD feature set can take less time. For the classification of knee and ankle movements in the sitting position, time consumption was 5.21 s (CSA) and 4.81 s (GOA); For the classification of movements in standing position, time consumption was 4.52 s (CSA) and 4.29 s (GOA). The TD + FD + WE feature set had the highest time consumption. For the classification of knee and ankle movements in the sitting position, time consumption was 10.21 s (CSA) and 9.00 s (GOA); For the classification of movements in the standing position, time consumption was 9.28 s (CSA) and 8.58 s (GOA).

In the sitting position, the confusion matrix of the four-class movements based on CSA-feature selection is shown as Table 2. The recognition rates of AD1 and AP1 were higher than 90% while that of KE1 and KF1 were comparatively lower: only slightly higher than 85%. A possible reason is that the distinction of muscle vibration between KE1 and KF1 was smaller, leading to lower feature-vector differences between the two class movements. When standing, the confusion matrix of movements based on CSA-feature selection is shown as Table 3. The recognition rate of KE2 was the lowest at 86.55%, while that of AD2 was the highest at 93.35%. Thus, both in sitting and standing, the recognition rates of KE and KF were lower than that for AD and AP.

### 3.2. Classification Results from Different Channel Combinations

**Figure 8:**
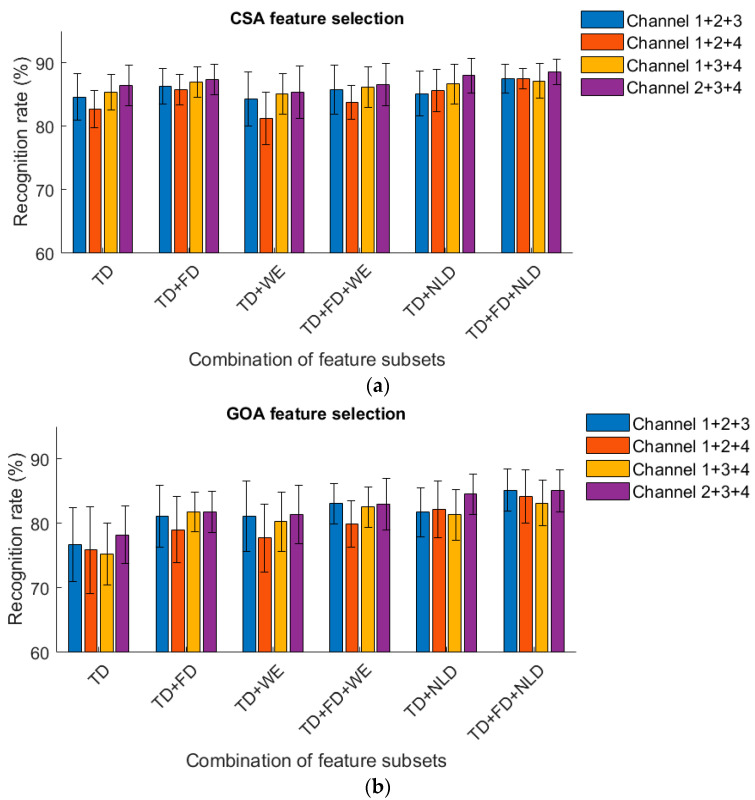
(**a**). Recognition rate obtained from 3-channel MMG (sitting, CSA). (**b**). Recognition rate obtained from 3-channel MMG (sitting, GOA).

**Figure 9:**
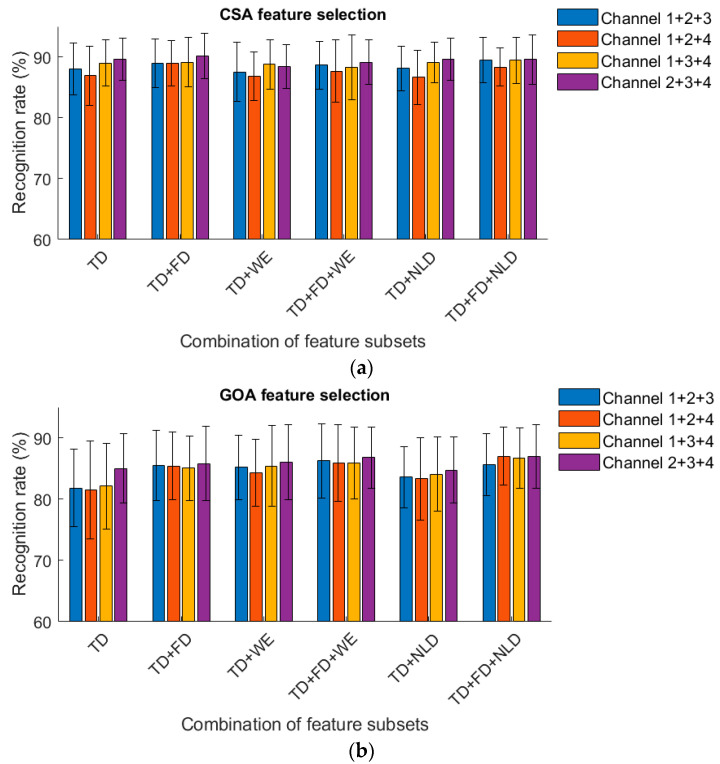
(**a**). Recognition rate obtained from 3-channel MMG (standing, CSA). (**b**). Recognition rate obtained from 3-channel MMG (standing, GOA).

For several 3-channel combinations, the recognition rates of knee and ankle movements based on CSA and GOA feature selection are shown in Figure 8 and Figure 9. Whether sitting or standing, using Channel 2 + 3 + 4 obtained the best classification results. When sitting, using CSA feature selection in the TD + FD + NLD feature set achieved a recognition rate of 88.56%. The recognition rates were 87.49% from Channel 1 + 2 + 3; 87.52% from Channel 1 + 2 + 4; and 87.14% from Channel 1 + 3 + 4. In the standing position, using CSA feature selection in the TD + FD + NLD feature set obtained a recognition rate of 89.56%. The recognition rates were 89.47% from Channel 1 + 2 + 3; 88.35% from Channel 1 + 2 + 4; and 89.42% from Channel 1 + 3 + 4. The results demonstrated that MMG from three muscles, i.e., vastus medialis, gastrocnemius lateralis and gastrocnemius medialis may have had a greater contribution to the recognition results than did vastus lateralis.

### 3.3. Classification Results from Different Threshold

**Figure 10:**
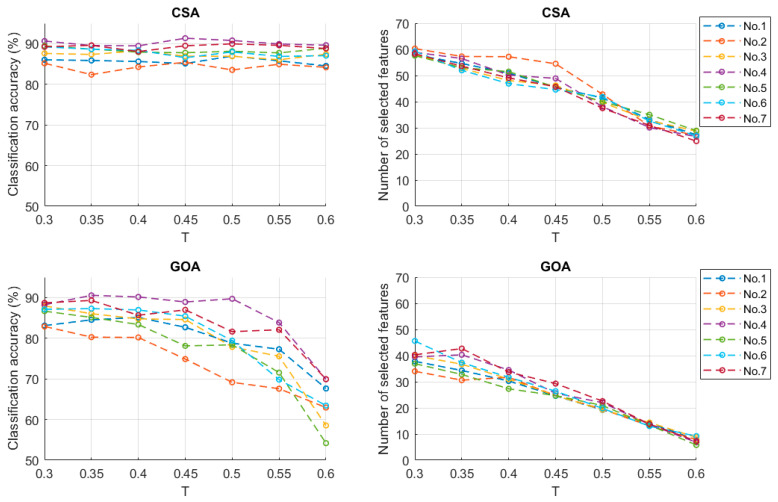
Classification accuracy and number of selected features (sitting).

In the sitting position, for a different threshold T, the relationship between classification accuracy and T values is shown as Figure 10. There were obvious distinctions between feature selection methods based on CSA and GOA. For the same T value, using CSA feature selection achieved higher accuracy than using the GOA, while the number of selected features from using the GOA was fewer. Taking 0.3 as an example, the average number of selected features by the CSA was 58.66 (sitting) and 59.03 (standing), while that of the GOA are 39.16 (sitting) and 44.27 (standing). When the value increased from 0.3 to 0.6, CSA classification accuracy for most of the subjects did not fluctuate much (the range values were 0.98) while that for the GOA decreased sharply: range value 25.58. There was no significant difference between the T value in 0.3 and 0.6 (*p* > 0.05) by using the CSA while the classification accuracy had a significant difference (*p* < 0.01) from using the GOA. For the two SIAs, the number of selected features decreased almost linearly as the threshold increased. When the threshold value was 0.35, the high accuracy of movement (in the standing situation) was 90.07%, and the average number of selected features was 53.26. When the threshold was 0.5, the average number of selected features (CSA) was 40.18 (sitting) and 38.67 (standing), and the classification results were 87.72% (sitting) and 88.75% (standing). When the threshold was 0.3, the average number of selected features (GOA) was 39.16 (sitting) and 44.27 (standing), and the classification results were 86.36% (sitting) and 89.33% (standing). Under these circumstances, the average number of selected features by the two intelligence algorithms were similar. By using feature selection methods based on CSA and GOA, the relationship between the recognition rates of each class movement and the threshold values is shown in Table 4 and Table 5. For the CSA, as the threshold increased from 0.3 to 0.6, the recognition rates of most class movements fluctuated slightly, though that of AP1 dropped from 90.40 to 87.90%. In contrast, for the GOA, the recognition rates of each class movements decreased sharply to lower than 70% when the threshold was 0.6.

**Figure 11:**
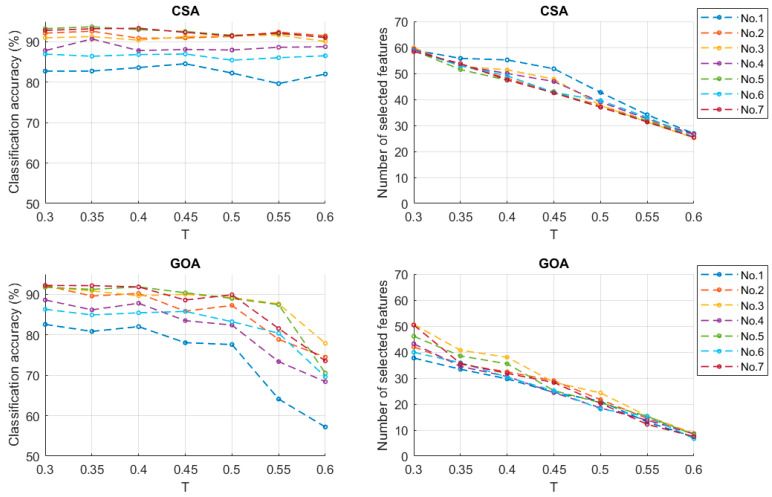
Classification accuracy and number of selected features (standing).

In the standing position, the relationship between classification accuracy and the T values is shown in Figure 11. Similar to sitting position, using CSA feature selection achieved better classification results than using the GOA; however the number of selected features from using the CSA was higher. By using feature selection methods based on CSA and GOA, the relationship between the recognition rates of each class movements and the threshold values are shown as Table 6 and Table 7. For the CSA, the recognition rates of most class movements fluctuated in a small range as the threshold increased (the range value is 1.37, *p* > 0.05). However, for the GOA, the recognition rates dropped to below 75%: range value 19.11 (*p* < 0.01) when the threshold value was 0.6. 

## 4. Discussion

For lower limb rehabilitation training, multichannel MMG signals were collected from the thigh, and feature selection methods based on two swarm intelligence algorithms were proposed for investigating pattern recognition of knee and ankle movement in sitting and standing positions, thereby demonstrating the relationship between classification accuracy and number of selected features. EMG sensors on the legs were adopted to represent the electrical activity of muscles during a lower limb movement [35]. Currently, studies about pattern recognition of lower limb movements based on sEMG, especially gait recognition are being applied to an exoskeleton [36]. Compared with sEMG, another biomedical signal, MMG is convenient for data collection, having investigative value for rehabilitation training system. Though MMG is different from sEMG in physical characteristics, both are enhanced and attenuated as muscles contract and relax, having some synchrony in static and transient states.

As an import part of biomedical-signal machine learning, feature extraction and selection directly affect the results of pattern recognition. The biomedical-signal features can be generally divided into TD, FD, TFD and NLD features, of which TD features are the most frequently adopted for sEMG or MMG classification problems. Because TD feature extraction is easy to compute and use it can achieve satisfactory classification results in many cases. In some other studies, the representative TD features, such as MAV, ZC, RMS, were used with high accuracy [37,38]. However, TD features alone may not use sufficient signal information; thus, sometimes they are combined with other features [39]. In this paper, though the TD features made a great contribution to MMG recognition rates of knee and ankle movements, using a TD + FD + NLD feature set achieved better results.

The design of the classifier is another important part in the studies about pattern recognition of gait movements based on sEMG. So far many scholars have applied various classification algorithms such as linear discriminant analysis [40], hidden Markov model [40], extreme learning machine [30] and SVM [33]. Khomami et al. [33] demonstrated that using an SVM with an RBF to build a classifier can achieve better sEMG classification results compared to other algorithms. In this paper, an SVM with an RBF were applied to be the classification algorithm of knee and ankle movements based on MMG. After feature extraction to remove the redundant information and improve applicability, feature transformation or selection were usually used to reduce the dimension [39], and the selected features reflected the difference between the patterns of samples. Currently, the traditional feature selection method ReliefF [21] has been used while a feature selection method based on swarm intelligence algorithms are reported comparatively less often. The CSA and GOA are two novel SIAs proposed in recent years, and it has been demonstrated that they have excellent performance in engineering optimization problems. MMG feature selection methods based on the CSA and GOA were proposed in this paper to investigate the pattern recognition of knee and ankle movements.

Although multiple signal channels can improve classification accuracy, fewer channels are more convenient. Thus, reducing the number of channels was meaningful for retaining high accuracy. According to the results from several three-channel combinations, using Channel 2 + 3 + 4 achieved the highest accuracy, approximating to using all four channels and higher than using the other three-channel combinations. It illustrated that, of the four thigh muscles, the vastus medialis, gastrocnemius lateralis and gastrocnemius medialis gave more positive classification results when the subjects performed the four-class knee and ankle movements. Regarding acquisition sites, collecting MMG from these three muscles gave better in classification results.

Muscle group synergy is a kind of neural control strategy that coordinates movements via the central nervous system. It can activate a muscle group according in different proportions. If muscle group synergy is considered with pattern recognition of lower limb movements, it is better for human physical mechanism. In the future, more experiments will be done to increase the number of samples, and deep learning combined with muscle group synergy will be used to investigate more applicable methods for lower limb movements.

## 5. Conclusions

In this paper, MMG feature selection methods based on the CSA and GOA were proposed for pattern recognition of knee and ankle movements. Of all the feature sets, TD + FD + NLD gave the best classification results. For the CSA-based feature-selection method, the classification accuracy of knee and ankle movements was 88.17% (sitting) and 90.07% (standing). Of all the three channel combinations, Channel 2 + 3 + 4 (vastus medialis, gastrocnemius lateralis and gastrocnemius medialis) made the most important contribution to accuracy. Concerning the same threshold, using CSA feature selection achieved higher accuracy than using the GOA, while the number of selected features by using the GOA was lower. For the CSA, recognition rates of most class movements fluctuated in a small range as the threshold increased. However, for the GOA, recognition rates decreased sharply when the threshold increased.

## Figures and Tables

**Table 1:** Formula of the transfer function.

Name	Formulation	Name	Formulation
S1	$\frac{1}{1+e^{-x}}$	V1	tanh(x)
S2	$\frac{1}{1+e^{-2x}}$	V2	$\frac{\left|x\right|}{\sqrt{1+x^{2}}}$
S3	$\frac{1}{1+e^{\left(-x/2\right)}}$		

**Table 2:** Confusion matrix of movements based on CSA-feature selection (sitting).

Targeted	Predicted Motion			
Motion	KE1	KF1	AD1	AP1
KE1	85.21	7.12	2.54	5.13
KF1	7.61	85.39	4.85	2.12
AD1	3.76	3.97	91.07	2.20
AP1	2.57	2.57	2.22	90.60

**Table 3:** Confusion matrix of movements based on CSA-feature selection (standing).

Targeted	Predicted Motion			
Motion	KE2	KF2	AD2	AP2
KE2	88.26	7.48	2.78	1.48
KF2	8.40	86.55	2.01	3.04
AD2	4.10	3.59	90.24	2.07
AP2	3.07	3.07	1.91	93.35

**Table 4:** Recognition rates of each movements in sitting situation (CSA).

	0.3	0.35	0.4	0.45	0.5	0.55	0.6
KE1	84.33	84.01	85.69	85.69	84.82	85.51	83.75
KF1	85.39	86.58	86.38	86.23	84.31	83.75	84.01
AD1	93.59	91.69	91.19	90.40	90.75	90.18	91.52
AP1	90.40	90.43	90.21	89.86	90.62	89.43	87.90

**Table 5:** Recognition rates of each movements in sitting situation (GOA).

	0.3	0.35	0.4	0.45	0.5	0.55	0.6
KE1	83.06	81.90	81.87	81.11	77.28	73.30	64.84
KF1	85.04	84.65	83.98	83.56	78.85	74.76	61.94
AD1	90.76	90.83	89.14	86.47	84.56	75.89	67.80
AP1	87.83	87.94	87.74	85.42	81.84	74.68	63.91

**Table 6:** Recognition rates of each movements in standing situation (CSA).

	0.3	0.35	0.4	0.45	0.5	0.55	0.6
KE2	87.80	87.27	85.62	87.38	86.45	86.50	85.41
KF2	87.56	86.72	88.02	87.44	87.57	87.11	85.70
AD2	90.08	89.47	91.02	90.00	89.63	90.27	89.72
AP2	93.09	94.05	92.20	92.70	93.54	93.44	92.28

**Table 7:** The recognition rates of each movements in standing situation (GOA).

	0.3	0.35	0.4	0.45	0.5	0.55	0.6
KE2	88.08	87.41	86.34	83.95	81.16	76.38	66.19
KF2	86.11	86.19	84.23	83.44	81.10	73.94	66.54
AD2	90.65	89.56	89.50	88.42	88.83	85.05	72.36
AP2	92.95	92.79	92.47	92.40	88.97	85.83	74.79
